# 

**Published:** 1889-05

**Authors:** 


					﻿Volume LVIII.
MAY, 1889.
Number 5'

fe Journal
EXAMINER
EDITOR :
S, J. JONES, M.D., LL.D.
PUBLISHED MONTHLY UNDER THt AUSPICES OF
THE CHICAGO MEDICAL PRESS ASSOCIATION.
Entered at the Post Office at Chicago, for transmission through the mails as second-class matter.
				

